# Supplementary material for: Upregulation of Transferrin and Major Royal Jelly Proteins in the Spermathecal Fluid of Mated Honeybee (Apis mellifera) Queens
Source: Insects. 2021 Jul 31;12(8):690. doi: 10.3390/insects12080690 (PMC8396679; doi:10.3390/insects12080690)
Supplement: Supplementary file 1 [file insects-12-00690-s001.zip › Table S1.pdf]

Table S1. The genes were differentially expressed in the spermathecae between mated queens and virgin queens.

Feature_GID	BEEBASE	Product	Fold change Mated queens/virgin queens	*p*-value
gene75	GB47662	D-galactonate transporter	-2.564208	4.8519E-05
gene86	GB47668	lipase 3-like	-2.144253	0.00099219
gene94	.	uncharacterized LOC107964453	-3.153919	5.9968E-06
gene95	GB47740	slit homolog 1 protein-like	-2.131095	7.9784E-05
gene98	.	uncharacterized LOC107964446	4.286483	0.00118273
gene99	.	uncharacterized LOC100578187	3.975287	0.00033166
gene100	.	uncharacterized LOC102655652	5.071412	0.00066779
gene101	.	uncharacterized LOC102655720	2.140501	0.00450706
gene102	.	uncharacterized LOC102655681	2.153250	0.00460862
gene105	.	uncharacterized LOC102655805	2.962847	9.843E-05
gene108	.	uncharacterized LOC102656241	11.606206	2.0354E-05
gene137	.	uncharacterized LOC107963981	2.648546	0.00175064
gene215	GB52266	furin-like protease 2	-2.447660	0.0003209
gene241	.	uncharacterized LOC102655293	2.225516	0.00427442
gene297	GB54868	phospholipase A1 member A	-2.432225	0.00011127
gene312	.	uncharacterized LOC100577822	2.926827	0.00403267
gene315	.	uncharacterized LOC102654454	3.218253	0.00023175
gene326	GB54851	zinc transporter 1	-3.113637	4.5134E-05
gene332	GB40947	sodium-dependent dopamine transporter-like	-3.491289	0.00074537
gene337	GB40944	A disintegrin and metalloproteinase with thrombospondin motifs 8	-2.581967	0.00053651
gene392	GB40769	farnesol dehydrogenase-like	-2.049116	1.5709E-05
gene448	GB40734	fructose-bisphosphate aldolase-like	2.090814	6.5319E-05
gene456	GB40838	endoglucanase E-4-like	2.628086	1.1548E-06
gene458	.	uncharacterized LOC107964593	2.818459	0.00061114
gene491	GB40864	titin-like	2.679627	0.00020807
gene504	GB40867	dopamine transporter	-3.197902	0.00127479
gene514	.	uncharacterized LOC102656814	-3.710508	0.0012693
gene529	GB51820	glucose dehydrogenase [FAD, quinone]	4.546175	6.6421E-05
gene536	.	uncharacterized LOC107965619	-2.144644	2.618E-05
gene538	GB51854	potassium voltage-gated channel subfamily KQT member 1-like	-2.827809	0.00057798
gene556	GB51841	multiple inositol polyphosphate phosphatase 1-like	-2.022092	3.5935E-07
gene562	GB48975	uncharacterized LOC100576961	-3.863469	0.00010368

gene563	GB48978	solute carrier family 22 member 8-like	-2.695607	8.1169E-07
gene646	GB41073	N-acetylgalactosamine kinase	2.163981	0.00027165
gene647	GB41074	uncharacterized LOC409078	-3.951845	0.01237122
gene648	GB41034	facilitated trehalose transporter Tret1-like	-2.202451	0.00313518
gene668	.	uncharacterized LOC102656299	2.658538	0.00018003
gene682	GB46547	uncharacterized LOC409701	-3.494821	0.00302267
gene695	GB45852	MAGUK p55 subfamily member 7	-2.851415	9.2563E-05
gene700	GB45850	clavesin-2	-3.459430	0.01151364
gene717	GB45834	pyruvate dehydrogenase E1 component subunit alpha, mitochondrial-like	-2.321978	0.00046421
gene718	GB45833	uncharacterized LOC413907	-3.534125	2.021E-05
gene751	GB45884	acyl-coenzyme A:6-aminopenicillanic-acid-acyltransferase 40 kDa form	-14.585273	2.3757E-06
gene778	GB44772	ATP-binding cassette sub-family G member 4-like	-2.416225	3.5512E-05
gene805	.	uncharacterized LOC102656816	9.396189	1.9134E-05
gene821	.	uncharacterized LOC100578133	2.137019	0.00016496
gene826	.	uncharacterized LOC100578110	2.736633	6.907E-05
gene835	GB44803	pyrimidodiazepine synthase	2.058953	1.6545E-05
gene836	.	uncharacterized LOC107964502	-2.073830	0.01497813
gene840	.	uncharacterized LOC107964509	2.115558	0.04164129
gene842	GB44808	peroxidasin	-3.382114	1.245E-05
gene848	.	farnesol dehydrogenase-like	2.025442	0.00268602
gene878	GB46518	uncharacterized LOC726920	-2.098033	0.01545546
gene894	.	uncharacterized LOC102655469	3.425346	0.00024837
gene896	.	uncharacterized LOC102655422	2.537559	0.00306184
gene897	GB46422	proton-coupled amino acid transporter 1	-2.415311	4.8765E-07
gene959	GB46468	uncharacterized LOC726722	2.118117	1.3763E-05
gene993	GB55280	serpin B3-like	2.152918	0.00063316
gene1019	GB47373	UPF0769 protein C21orf59 homolog	-2.046222	1.7383E-05
gene1026	GB47379	histidine decarboxylase	-2.115511	0.00035394
gene1029	.	histone H3	2.628803	0.0028924
gene1031	.	histone H2B-like	2.204490	0.00271175
gene1036	GB47503	delta-1-pyrroline-5-carboxylate synthase	-2.825125	1.2994E-06
gene1069	.	histone H3	2.215801	0.00055832
gene1081	GB47478	glutathione peroxidase-like 1	2.072996	0.04964722
gene1087	GB47475	protein lethal(2)essential for life-	2.656032	7.8979E-06

		like		
gene1108	GB47463	guanine nucleotide-binding protein G(i) subunit alpha	2.708939	0.00248651
gene1140	GB49726	thrombospondin type-1 domain-containing protein 4-like	-3.909972	0.00011542
gene1148	GB49732	rho guanine nucleotide exchange factor 17	-2.338713	1.4086E-05
gene1159	GB51938	leucine rich repeat G protein coupled receptor	-2.544625	8.5772E-05
gene1219	GB42196	thrombospondin type-1 domain-containing protein 4-like	-2.558696	3.6607E-06
gene1258	GB42220	electroneutral sodium bicarbonate exchanger 1	-2.067893	0.00026891
gene1261	.	acyl-CoA Delta(11) desaturase-like	3.344400	0.0089205
gene1262	GB42218	acyl-CoA Delta(11) desaturase	2.647423	5.4382E-05
gene1263	.	acyl-CoA desaturase-like	3.145868	2.3637E-05
gene1331	.	uncharacterized LOC107966053	3.025765	6.4862E-05
gene1342	GB51591	3-hydroxy-3-methylglutaryl-coenzyme A reductase	-3.274753	0.00025859
gene1356	GB51583	kynurenine/alpha-aminoadipate aminotransferase, mitochondrial-like	-2.157992	5.7745E-05
gene1432	GB51636	ribonuclease kappa-B	-2.010947	0.00010524
gene1459	.	uncharacterized LOC107966045	-3.337933	6.6688E-05
gene1519	.	microRNA 6053	5.653627	0.00018634
gene1535	GB54988	mitochondrial sodium/hydrogen exchanger 9B2-like	-2.674213	0.00155006
gene1544	.	probable RNA methyltransferase CG11342	2.062047	0.02059464
gene1545	.	tribbles homolog 2	2.044614	3.4432E-05
gene1568	GB13325	chemosensory protein 6	2.217890	0.00017506
gene1578	.	uncharacterized LOC107965571	-6.730173	0.00560413
gene1610	GB53925	uncharacterized LOC724993	3.764129	0.00012167
gene1666	.	myb-related transcription factor, partner of profilin-like	2.957300	0.00014218
gene1689	.	uncharacterized LOC100577437	2.380166	0.00048221
gene1726	GB50700	fibrillin-1-like	-2.337067	0.00411434
gene1739	GB46311	cuticular protein 21	-2.330789	0.00083989
gene1740	GB46312	cuticular protein 22	-2.739141	0.00240793
gene1742	GB46297	cuticular protein 14	-2.136552	0.00131358
gene1743	.	uncharacterized LOC107964094	2.209405	0.02907987
gene1785	.	ceramide phosphoethanolamine synthase	2.411391	7.1883E-06
gene1803	GB43455	uncharacterized LOC100578397	21.100940	0.00560237
gene1807	GB43453	band 4.1-like protein 4A	-2.165238	5.4067E-05

gene1810	GB45906	uncharacterized LOC410087	4.680952	2.3618E-06
gene1812	GB45910	protein lethal(2)essential for life-like	34.151509	2.5896E-05
gene1813	.	protein lethal(2)essential for life-like	18.387163	1.5957E-05
gene1814	GB45912	protein lethal(2)essential for life-like	3.107037	1.3465E-06
gene1829	.	uncharacterized LOC107964083	11.838154	1.5816E-05
gene1830	GB50772	uncharacterized LOC100578420	2.752550	0.00015681
gene1893	GB50831	protein canopy homolog 2	-3.430001	5.5847E-05
gene1974	.	probable salivary secreted peptide	-4.134599	0.00338716
gene2002	.	uncharacterized LOC102656216	3.448415	0.00197415
gene2036	.	uncharacterized LOC726803	2.682654	0.00025727
gene2041	GB52467	uncharacterized LOC408695	-2.788808	0.00112627
gene2135	.	uncharacterized LOC107966102	4.267583	0.00636244
gene2143	GB52497	mitochondrial import receptor subunit TOM20 homolog	-2.424779	0.00036019
gene2159	GB52343	neurotrimin	2.857148	3.6736E-08
gene2180	.	uncharacterized LOC102653599	4.861269	1.1183E-07
gene2181	GB49775	protein lethal(2)essential for life-like	3.460410	0.00056708
gene2202	.	uncharacterized LOC102654905	-2.256845	0.00013953
gene2215	GB49809	calmodulin-like	-3.529398	9.2871E-05
gene2273	.	cofilin/actin-depolymerizing factor homolog	2.201828	0.00035568
gene2286	GB55638	tryptophan 2,3-dioxygenase	-4.041994	0.00010655
gene2310	.	uncharacterized LOC102653583	2.002765	0.0002512
gene2320	.	protein THEM6-like	-2.774529	0.00017611
gene2326	GB55613	uncharacterized LOC100576118	-4.722262	1.2405E-05
gene2327	GB55612	uncharacterized LOC552320	-28.728309	1.3415E-06
gene2329	GB55396	uncharacterized LOC552356	-3.094109	0.00016415
gene2330	.	microRNA 6060	-2.958942	0.00030233
gene2334	GB55403	uncharacterized LOC410819	-2.558818	5.0383E-05
gene2355	GB55598	troponin I	-2.783162	5.656E-06
gene2371	GB55426	uncharacterized LOC725893	3.104666	0.00015581
gene2379	GB55591	titin	2.331660	5.921E-05
gene2420	GB55443	endoplasmic reticulum resident protein 29	-3.162470	4.0016E-07
gene2427	.	uncharacterized LOC102656130	2.433459	6.949E-06
gene2442	GB55561	copper transport protein ATOX1	-2.220398	0.00020377
gene2464	GB55547	chemosensory protein 2	-2.001815	0.00021499
gene2502	.	uncharacterized LOC107966105	3.165179	9.13E-05
gene2514	GB55516	bone morphogenetic protein 2-B	2.976143	2.6483E-05

gene2515	GB55515	inositol oxygenase	3.377126	3.4625E-05
gene2524	GB55499	alkaline phosphatase 4-like	-16.088831	1.1394E-06
gene2525	.	uncharacterized LOC107963987	-9.647610	4.7053E-05
gene2526	.	uncharacterized LOC100577377	-11.810446	8.9611E-05
gene2528	GB55503	solute carrier family 41 member 1-like	2.799691	1.1431E-05
gene2545	GB55998	beta-1,4-N-acetylgalactosaminyltransferase bre-4	-2.192636	2.0727E-05
gene2547	GB55997	low density lipoprotein receptor adapter protein 1-B-like	-3.996433	1.3529E-05
gene2551	GB56017	paired mesoderm homeobox protein 2-like	-6.691733	0.00010365
gene2554	GB51881	uncharacterized LOC100577971	6.787772	1.1756E-06
gene2556	.	uncharacterized LOC100577932	4.704217	0.00012845
gene2602	.	uncharacterized LOC102654462	4.450712	6.1315E-05
gene2613	.	uncharacterized LOC107964144	2.275340	0.00049741
gene2614	.	uncharacterized LOC102654823	2.105173	0.00252327
gene2670	.	uncharacterized LOC102654687	2.241979	0.0231318
gene2671	GB49054	uncharacterized LOC410975	2.616080	5.3665E-05
gene2736	.	uncharacterized LOC100576853	2.357625	4.4456E-05
gene2744	.	uncharacterized LOC100577278	-2.182232	0.0041316
gene2747	.	uncharacterized LOC100577303	2.210966	0.00485772
gene2754	GB46962	SH3 domain-binding glutamic acid-rich protein homolog	2.181002	4.7824E-05
gene2792	GB51487	proton-coupled amino acid transporter 1-like	2.214671	0.00019118
gene2835	GB55813	uncharacterized LOC100577773	-6.291073	0.00052742
gene2871	GB55767	cGMP-dependent protein kinase 1-like	-2.388436	0.0001699
gene2899	GB55846	kinesin 12	2.339830	4.1066E-05
gene3038	.	uncharacterized LOC107964193	3.397421	5.3346E-06
gene3039	.	uncharacterized LOC100576419	2.429342	0.00186758
gene3059	GB53835	zinc finger protein 395	-2.030538	5.7022E-06
gene3090	GB49184	PH domain leucine-rich repeat-containing protein phosphatase 2	2.186911	0.0001633
gene3101	.	uncharacterized LOC102656502	2.987246	0.00182787
gene3125	GB47055	putative inorganic phosphate cotransporter	12.828862	1.8182E-05
gene3131	.	uncharacterized LOC102654960	-2.270224	0.00030958
gene3150	.	uncharacterized LOC107964139	2.154922	0.00090472
gene3151	.	uncharacterized LOC100577718	2.298350	0.00041932
gene3184	GB41203	cuticular protein analogous to peritrophins 3-C	-2.623140	2.9002E-05
gene3225	GB41223	uncharacterized LOC100578584	2.443592	0.00126982

gene3236	.	uncharacterized LOC100578320	2.993226	3.4484E-05
gene3237	GB41228	uncharacterized LOC724900	3.217730	0.00091358
gene3266	GB46142	sarcoplasmic calcium-binding protein	5.529534	7.3252E-05
gene3313	GB49639	putative defense protein 3	-2.043354	0.01069059
gene3349	GB53119	apidermin 2	-2.526320	0.00042838
gene3351	GB53114	apidermin 3	-2.075275	0.04423855
gene3364	.	transcriptional regulator ATRX homolog	-3.874316	1.1675E-07
gene3365	GB53104	cubilin	-2.472835	0.00036778
gene3391	GB53139	testican-1	-2.127334	0.00038837
gene3517	GB49543	alanine--glyoxylate aminotransferase 2-like	3.792303	3.628E-05
gene3518	GB49544	vitellogenin	6.160663	6.659E-06
gene3523	.	uncharacterized LOC102656496	12.583079	5.9593E-06
gene3524	.	uncharacterized LOC107964285	13.748905	1.0015E-05
gene3635	GB50438	cuticular protein 27	-2.862854	0.00182424
gene3639	GB50449	uncharacterized LOC100578672	-6.713950	1.6054E-05
gene3641	GB50450	bromodomain-containing protein 4-like	-5.780669	8.7189E-05
gene3663	GB50423	uncharacterized LOC408807	6.134058	7.6462E-05
gene3731	GB42994	tensin-1	-6.096421	3.6622E-06
gene3750	.	uncharacterized LOC102655336	-7.544994	7.8218E-05
gene3782	.	uncharacterized LOC726866	2.525247	5.5729E-05
gene3783	GB44112	melittin	-7.664072	4.1585E-06
gene3810	GB44080	protein-L-isoaspartate(D-aspartate) O-methyltransferase-like	2.362317	7.5435E-06
gene3824	GB44133	tubulin beta-1	2.112561	0.00122761
gene3828	GB44074	tubulin beta chain	-2.144658	0.00269789
gene3904	.	uncharacterized LOC107964318	2.568308	0.0180489
gene3935	.	uncharacterized LOC725381	9.651677	9.5156E-06
gene3952	GB44213	filamin-like	4.301521	1.8757E-05
gene3968	GB44005	sialin-like	2.243046	1.4972E-05
gene3979	.	uncharacterized LOC100576929	-2.115087	0.0001183
gene3994	GB53015	protein FAM151B	3.897279	1.9998E-05
gene3995	.	DNA ligase 1-like	2.170933	0.00024609
gene4091	GB52279	neurexin 1	-3.235286	0.00104212
gene4092	.	secapin	-13.812339	9.8897E-08
gene4093	.	uncharacterized LOC102655788	-27.103996	7.7421E-07
gene4100	GB52324	chemosensory protein 3	-2.141367	0.00016245
gene4106	.	uncharacterized LOC107964518	-2.694538	3.6265E-06

gene4114	GB48871	DNA polymerase epsilon subunit 2	2.468588	0.00653441
gene4199	GB46726	proclotting enzyme	-2.012130	0.00112979
gene4230	GB46703	synaptonemal complex protein ZEP1-like	2.291103	0.00037861
gene4251	GB46774	dnaJ protein homolog 1	2.023128	0.00038995
gene4284	.	uncharacterized LOC102655605	4.604567	0.00034754
gene4286	.	uncharacterized LOC102655278	2.589883	0.00133362
gene4296	.	uncharacterized LOC100576657	2.962831	6.4988E-07
gene4390	.	uncharacterized LOC102654470	2.164638	0.00297721
gene4444	GB50302	RNA-binding protein 34-like	2.037269	3.6459E-06
gene4456	.	uncharacterized LOC725148	-3.856580	0.00017563
gene4463	GB44564	protein NPC2 homolog	-2.302471	0.00022682
gene4466	GB44548	glucose dehydrogenase [FAD, quinone]	4.022667	9.8136E-06
gene4515	GB44596	uncharacterized LOC409510	2.356587	0.00072616
gene4528	GB44513	cytochrome P450 4c3	-2.257312	4.8448E-06
gene4529	GB44607	uncharacterized LOC100578566	-2.313572	3.536E-05
gene4543	GB44503	uncharacterized LOC727423	4.165467	1.0057E-05
gene4548	.	uncharacterized LOC107964500	3.875281	0.00199476
gene4552	GB44616	EF-hand domain-containing family member C2-like	8.815590	0.00014018
gene4555	GB44494	arylsulfatase B-like	-2.320036	0.00019883
gene4556	GB44493	arylsulfatase B-like	7.359991	2.5987E-07
gene4559	GB44491	nucleoside-triphosphatase THEP1	-2.349434	0.00011762
gene4566	.	uncharacterized LOC102656464	-2.299786	0.00119109
gene4568	.	uncharacterized LOC107964475	3.805515	0.00738676
gene4569	.	uncharacterized LOC100576597	2.466153	0.00097136
gene4581	.	uncharacterized LOC102654490	-2.003769	0.01448566
gene4603	.	uncharacterized LOC107963997	2.751844	0.00199041
gene4665	GB44430	dihydrolipoyllysine-residue succinyltransferase component of 2-oxoglutarate dehydrogenase complex, mitochondrial-like	-2.744234	0.00140685
gene4685	GB44419	septin-7	2.262664	7.6698E-05
gene4702	GB44412	hemicentin-2	-3.163507	9.1369E-07
gene4707	GB44708	odorant receptor 13a-like	2.574165	0.00094813
gene4715	GB47849	pyrroline-5-carboxylate reductase 2	-3.029100	8.8643E-08
gene4716	GB47814	transmembrane protein 135-like	-7.421561	9.9247E-05
gene4718	GB47815	haloacid dehalogenase-like hydrolase domain-containing protein 2	-2.451360	0.00062708
gene4756	GB41945	uncharacterized LOC726189	5.601916	3.3032E-05

gene4757	.	uncharacterized LOC102656699	5.766141	0.00089865
gene4762	GB41376	J domain-containing protein	2.055446	1.1911E-05
gene4767	.	waprin-Thr1	2.347957	3.8164E-06
gene4771	GB41289	uncharacterized LOC411664	5.392076	0.02246337
gene4772	.	uncharacterized LOC102656586	5.956198	9.113E-05
gene4811	GB41301	annexin B9-like	2.546501	0.00015721
gene4824	.	actin, clone 205-like	-2.982343	5.8953E-05
gene4825	.	uncharacterized LOC102654094	-2.991135	0.00489641
gene4826	.	actin, clone 205-like	-2.467784	0.00019049
gene4828	GB41310	actin, muscle-like	-2.658652	0.00039274
gene4842	GB41325	uncharacterized LOC100578835	3.064330	0.00020114
gene4843	.	uncharacterized LOC100577646	2.033151	0.00053994
gene4844	.	uncharacterized LOC100577107	3.936892	0.00105704
gene4860	GB45633	uncharacterized LOC726990	2.943623	0.00010427
gene4885	GB45650	uncharacterized aarF domain-containing protein kinase 2-like	2.713077	0.00081739
gene4891	GB45654	gamma-glutamyltranspeptidase 1-like	-2.499407	6.9601E-06
gene4911	.	uncharacterized LOC102654951	-2.033322	0.03757235
gene4915	GB45700	serine protease easter	3.059854	0.00033508
gene4927	GB45692	dentin sialophosphoprotein	-2.336451	0.00011728
gene4928	GB45673	alpha-N-acetylgalactosaminidase	-4.595871	1.0792E-05
gene4932	GB45688	synaptic vesicle membrane protein VAT-1 homolog-like	2.234828	1.324E-05
gene4949	GB51188	lysophospholipid acyltransferase 2	2.589642	3.5589E-06
gene4960	GB51214	troponin T, skeletal muscle	-3.198362	5.4491E-05
gene4977	GB51200	DNA-binding protein D-ETS-6-like	3.479310	9.5048E-05
gene4980	GB45771	glycine-rich RNA-binding protein 7	-25.982012	4.5139E-05
gene4998	.	uncharacterized LOC726672	2.415772	0.00020969
gene5003	GB48078	beta-1,4-glucuronyltransferase 1	-3.434570	2.8568E-05
gene5006	.	uncharacterized LOC102656738	-3.333378	2.096E-07
gene5038	GB54485	UDP-glucuronosyltransferase 1-3-like	25.221516	7.5989E-07
gene5040	GB54486	myrosinase 1-like	-11.439247	2.8381E-05
gene5041	.	uncharacterized LOC107964635	-18.978242	0.0007752
gene5042	GB54460	uncharacterized LOC725017	-27.563506	2.9099E-09
gene5046	GB54454	uncharacterized LOC724783	2.084741	0.00272226
gene5047	.	uncharacterized LOC107964657	-2.170219	0.00029601
gene5069	GB43247	alpha-glucosidase	5.173671	2.4769E-05
gene5071	GB43246	uncharacterized protein KIAA0513	-2.113333	4.4307E-06

gene5092	GB52191	coagulation factor X	2.709216	6.1636E-07
gene5100	GB52161	cuticular protein 28	-25.593781	1.9539E-09
gene5101	GB52199	uncharacterized LOC100578593	3.116975	0.00213543
gene5102	.	uncharacterized LOC102655655	2.931152	0.00017756
gene5176	GB46015	probable cytochrome P450 301a1, mitochondrial	-2.282413	0.00270119
gene5177	GB46062	probable cytochrome P450 301a1, mitochondrial	2.585486	0.00094964
gene5200	GB46047	cilia- and flagella-associated protein 70	-2.163129	3.3869E-05
gene5210	GB46039	tubulin alpha-1 chain-like	3.985183	8.1926E-06
gene5214	GB46030	cys-loop ligand-gated ion channel subunit 12344	-2.433674	0.00015721
gene5219	.	uncharacterized LOC100577517	-3.051288	6.3994E-07
gene5221	.	uncharacterized LOC107964572	-4.805130	0.00017276
gene5264	.	uncharacterized LOC102654275	2.200757	0.00296546
gene5267	.	uncharacterized LOC107964547	2.603646	0.00168571
gene5279	GB48552	mesencephalic astrocyte-derived neurotrophic factor homolog	-2.461108	0.00057829
gene5280	GB48576	sushi, von Willebrand factor type A, EGF and pentraxin domain-containing protein 1-like	-2.379942	0.00024493
gene5287	GB48573	probable multidrug resistance-associated protein lethal(2)03659	-2.038112	6.0261E-05
gene5288	.	uncharacterized LOC107964602	-2.033948	0.00079471
gene5292	.	uncharacterized LOC107964603	3.161602	3.8136E-05
gene5335	.	uncharacterized LOC100578409	3.977084	2.6788E-06
gene5340	GB41427	catalase	2.777956	2.6476E-06
gene5362	.	uncharacterized LOC102654087	2.127095	1.784E-05
gene5370	GB41495	sarcalumenin	-2.735264	0.00053204
gene5406	.	uncharacterized LOC102655393	2.941139	1.456E-05
gene5415	.	protein mab-21	7.805535	3.9919E-06
gene5455	.	uncharacterized LOC102656909	2.258139	3.6195E-05
gene5506	GB43409	oocyte zinc finger protein XlCOF8.4-like	2.008260	0.01365486
gene5530	.	uncharacterized LOC107964540	2.853806	0.00017616
gene5531	GB53565	endochitinase	-4.251362	0.00054833
gene5535	GB53567	branched-chain-amino-acid aminotransferase, cytosolic-like	-2.914179	2.6339E-06
gene5569	GB53579	putative glucosylceramidase 4	-2.278800	1.2024E-05
gene5641	GB45376	putative peptidyl-prolyl cis-trans isomerase dodo	2.262796	5.7378E-05
gene5756	.	uncharacterized LOC102655018	3.179953	2.3804E-06
gene5760	.	uncharacterized LOC107964673	2.529864	6.9666E-06
gene5765	GB45464	putative leucine-rich repeat-	2.650881	0.00064174

		containing protein DDB_G0290503		
gene5858	GB52877	uncharacterized LOC726699	-2.016380	6.3971E-05
gene5892	GB49700	facilitated trehalose transporter Tret1-like	2.240923	0.00010105
gene5909	GB44265	uncharacterized LOC100576687	5.602897	1.5526E-05
gene5952	GB46276	apolipoprotein D-like	-2.218468	0.00427663
gene5958	GB42567	venom dipeptidyl peptidase 4-like	-2.647022	0.00703827
gene5979	.	protein takeout-like	-3.757935	1.8553E-07
gene5987	GB42550	mitochondrial amidoxime-reducing component 1-like	-6.479208	4.2929E-07
gene5990	.	uncharacterized LOC107964756	2.057614	0.00449195
gene5992	.	uncharacterized LOC102654659	2.103421	3.4498E-05
gene6022	.	uncharacterized LOC100577571	9.533861	0.00048738
gene6023	GB53798	esterase E4-like	7.452972	4.6841E-05
gene6024	GB53775	retinol dehydrogenase 14	4.469145	0.00043984
gene6037	GB53805	uncharacterized protein DDB_G0272420-like	2.707648	9.9676E-05
gene6051	GB49240	aldehyde dehydrogenase, mitochondrial	-2.511599	0.00059239
gene6090	GB43298	loricrin-like	-6.631094	1.7843E-05
gene6106	GB43309	uncharacterized LOC100576528	-2.037006	0.00547432
gene6135	.	uncharacterized LOC102655685	2.141516	0.00011653
gene6138	GB47974	carboxylesterase	-2.454580	0.00423599
gene6166	GB40976	heat shock protein 90	3.159889	1.6158E-05
gene6171	.	facilitated trehalose transporter Tret1-like	-3.086859	0.0001201
gene6173	.	ras-related and estrogen-regulated growth inhibitor	-2.552357	0.00019261
gene6175	GB40967	tyrosine hydroxylase	-3.771429	1.7429E-05
gene6210	GB42487	calpain-C	2.250530	1.6427E-05
gene6217	.	organic cation transporter protein	-3.347542	0.00834589
gene6230	GB42469	phospholipase B1, membrane-associated-like	-4.372407	6.8849E-06
gene6231	GB42468	phospholipase B1, membrane-associated-like	-2.345035	6.8608E-05
gene6232	GB42467	phospholipase B1, membrane-associated-like	-3.321052	3.332E-05
gene6287	GB44294	uncharacterized LOC100579008	2.615971	0.00023572
gene6289	GB44367	phospholipase A2-like	-9.382436	2.7094E-05
gene6293	.	uncharacterized LOC102655868	2.380593	3.8659E-05
gene6324	GB44311	actin related protein 1	3.542475	0.00019024
gene6347	.	histone-lysine N-methyltransferase SETMAR-like	-4.071479	7.6928E-06
gene6416	GB48125	myosin-10	-3.430120	7.7932E-06

gene6430	GB48147	uncharacterized LOC100578269	3.114942	0.00062683
gene6436	GB48134	L-lactate dehydrogenase-like	14.025383	4.9111E-07
gene6458	.	uncharacterized LOC102654281	-2.193777	0.0001326
gene6476	.	uncharacterized LOC102656347	2.278664	0.00816875
gene6525	GB41163	RWD domain-containing protein 2A	2.021294	2.2064E-05
gene6563	GB41126	synaptic vesicle glycoprotein 2C-like	-6.544206	0.00018636
gene6577	.	pro-resilin-like	-46.722745	0.00018686
gene6578	.	cuticular protein 8	-2.590800	0.00024665
gene6583	GB40451	alanine aminotransferase 1	3.249913	3.1523E-06
gene6584	GB40442	cysteine sulfinic acid decarboxylase	2.289774	7.0696E-05
gene6589	GB40438	uncharacterized LOC724393	2.232474	0.00105127
gene6604	GB40425	uncharacterized LOC727649	-2.521193	0.00125275
gene6621	.	uncharacterized LOC102653729	2.202832	0.0004429
gene6658	.	uncharacterized LOC102656154	2.472142	9.3915E-05
gene6723	GB40548	myosin heavy chain, non-muscle-like	2.403737	0.00092702
gene6745	GB40343	uncharacterized LOC413620	-2.569486	0.00360616
gene6759	GB40337	pyrokinin-like receptor 1	-2.342075	0.00011245
gene6761	GB40336	neutral ceramidase	-2.373927	0.00052206
gene6773	GB54519	uncharacterized LOC100577080	-2.251793	0.00024397
gene6774	GB54518	uncharacterized LOC100577110	-2.150727	0.00166191
gene6781	GB54538	uncharacterized LOC411248	-8.030113	0.00043295
gene6793	GB54506	uncharacterized LOC411253	2.334122	5.6186E-05
gene6803	.	uncharacterized LOC102656871	32.426704	1.0371E-09
gene6847	GB43909	plasma membrane calcium-transporting ATPase 2	2.319811	2.7212E-05
gene6856	GB43823	chemosensory protein 1	6.442684	0.00015308
gene6890	GB43892	cytosolic 10-formyltetrahydrofolate dehydrogenase	-3.947472	3.7944E-06
gene6896	GB43847	doublesex- and mab-3-related transcription factor A2-like	-2.103741	0.00246578
gene6919	GB43877	aquaporin-like	-2.179909	0.00030363
gene6939	GB43862	uncharacterized LOC100577444	2.372326	0.00497101
gene6940	.	uncharacterized LOC102654944	3.025725	0.00169346
gene6963	GB41756	GB15078 protein	2.192376	0.00981783
gene6964	GB41757	immunoglobulin domain-containing protein oig-1-like	2.128232	0.00655322
gene6972	GB41760	lipase 3-like	-15.389973	0.00017827
gene7011	.	probable uridine nucleosidase 2	-2.200220	0.00014567
gene7027	.	uncharacterized LOC107964906	4.023462	4.2494E-05

gene7029	GB41792	uncharacterized protein DDB_G0287625-like	2.376118	2.7265E-05
gene7051	GB41806	calcyphosin-like protein	2.356061	0.00011466
gene7079	GB41855	patched domain-containing protein 3	2.125340	2.2891E-05
gene7092	GB41844	ATP-binding cassette sub-family G member 5	2.757558	5.4545E-06
gene7107	GB41833	uncharacterized LOC100576880	-2.108939	0.0089754
gene7108	GB52819	uncharacterized LOC551572	-3.319770	1.1877E-06
gene7110	GB52854	cuticular protein analogous to peritrophins 3-E	-2.730426	3.2856E-05
gene7112	GB52824	pro-resilin	-2.136907	0.00353176
gene7119	.	uncharacterized LOC102655947	-2.993078	0.00018441
gene7184	GB48851	myosin heavy chain 95F	-2.398580	4.07E-05
gene7185	GB48850	fatty-acid amide hydrolase 2-B-like	-6.436911	0.00061067
gene7211	GB41979	UTP--glucose-1-phosphate uridylyltransferase	2.213768	8.4812E-05
gene7238	GB42000	cAMP-specific 3',5'-cyclic phosphodiesterase	2.380219	3.2857E-05
gene7241	GB41965	uncharacterized LOC100576936	2.304946	0.00034111
gene7263	GB51697	hexamerin 70b	-2.075809	0.00682758
gene7265	GB51696	hexamerin 70c	-2.311419	4.9993E-06
gene7290	GB51729	4-coumarate--CoA ligase 1-like	-2.349443	0.00032978
gene7293	GB51732	calcium and integrin-binding protein 1-like	3.525198	3.4721E-06
gene7301	GB51736	tweedle motif cuticular protein 2	3.836771	0.00022627
gene7317	GB51741	peptidoglycan recognition protein S3	2.020008	0.00079545
gene7332	GB51656	zinc finger HIT domain-containing protein 3	-2.683185	1.233E-05
gene7341	GB51653	myosin heavy chain, muscle	-2.220551	0.00012218
gene7369	GB45538	fructose-1,6-bisphosphatase 1	2.441943	1.3296E-05
gene7384	GB47140	uncharacterized LOC409465	4.802541	1.0161E-06
gene7386	GB47138	calcium-activated potassium channel slowpoke	-2.138337	4.439E-05
gene7391	GB47136	uncharacterized LOC724177	2.240035	0.00010027
gene7392	.	uncharacterized LOC102654076	2.264333	5.5202E-05
gene7395	GB41403	lachesin-like	-3.097082	0.00018562
gene7397	GB41406	uncharacterized LOC413274	-2.466960	2.0398E-05
gene7409	.	glycerol-3-phosphate dehydrogenase [NAD(+)], cytoplasmic-like	-7.832446	2.1015E-05
gene7485	.	uncharacterized LOC102655174	-2.196754	0.00305232
gene7495	.	myb/SANT-like DNA-binding domain-containing protein 3	2.125386	0.00224937

gene7543	GB42787	dentin sialophosphoprotein-like	-2.151930	0.00099058
gene7546	GB42792	uncharacterized LOC409805	-2.380099	0.00175021
gene7554	GB42704	protein takeout-like	-43.542537	3.7655E-07
gene7558	GB42797	circadian clock-controlled protein	-4.178508	4.6525E-05
gene7560	GB42798	protein takeout-like	-6.141341	8.6092E-05
gene7561	GB42702	circadian clock-controlled protein-like	-64.759207	5.9036E-05
gene7562	GB42701	uncharacterized LOC100576902	-2.456413	0.00121434
gene7563	GB42799	protein takeout-like	-24.764958	3.4855E-06
gene7564	GB42800	uncharacterized LOC727028	11.492256	0.00023038
gene7565	.	MFS-type transporter SLC18B1-like	5.675346	2.4589E-06
gene7566	GB42802	MFS-type transporter SLC18B1-like	6.254687	1.0775E-05
gene7577	GB42693	la protein homolog	2.922912	0.00030599
gene7590	.	uncharacterized LOC724583	-10.363591	0.00013033
gene7613	GB42673	retinol dehydrogenase 10-A-like	3.734285	0.00291791
gene7673	GB42634	mitochondrial folate transporter/carrier	2.043926	2.8875E-05
gene7678	.	uncharacterized protein C11orf70 homolog	2.135214	0.03132662
gene7715	GB42612	pupal cuticle protein 20	-7.928902	0.00010683
gene7745	GB42596	CG1567-like	-2.992161	0.00082685
gene7748	GB42594	organic solute transporter alpha-like protein	-2.464993	0.00048591
gene7749	.	inner centromere protein A	-2.492674	2.0634E-06
gene7770	GB42581	cuticular protein 11	-2.494665	0.00203893
gene7771	GB42580	D-beta-hydroxybutyrate dehydrogenase, mitochondrial	-2.586635	0.00063591
gene7773	.	uncharacterized LOC100576161	-42.515252	0.00040273
gene7790	.	microRNA 9889	2.076709	0.01497337
gene7820	GB53371	odorant binding protein 3	-2.227137	0.00727453
gene7847	.	chymotrypsin-like protease CTRL-1	-5.109383	9.1683E-06
gene7875	GB53414	serine/threonine-protein kinase ICK	-3.111528	1.8993E-05
gene7914	GB53428	nicotinic acetylcholine receptor beta2 subunit	-2.314940	0.00033027
gene7926	GB53434	p21-activated protein kinase-interacting protein 1-like	2.074931	0.00076006
gene7927	GB53300	endothelin-converting enzyme 1-like	-2.584253	0.00081318
gene7962	GB56028	uncharacterized LOC100576134	2.403120	0.0005235
gene7968	GB47918	netrin receptor UNC5C	-3.319316	9.6408E-05
gene7981	.	uncharacterized LOC102656324	2.038131	3.4394E-05
gene8021	GB46369	cytochrome c1, heme protein,	-2.238272	0.00929497

		mitochondrial		
gene8059	GB55889	matrix metalloproteinase-14	2.776308	0.00046926
gene8062	GB55888	kinesin 3A	-2.196434	8.3565E-05
gene8166	GB48382	solute carrier family 22 member 21-like	-2.984152	7.4247E-06
gene8168	GB48380	major facilitator superfamily domain-containing protein 8	-2.143459	7.7175E-05
gene8194	GB48364	inactive hydroxysteroid dehydrogenase-like protein 1	-2.427318	0.00022364
gene8195	GB48419	carbohydrate sulfotransferase 11-like	-2.486161	3.3711E-05
gene8212	.	uncharacterized LOC100578455	2.602926	0.00070076
gene8216	GB48350	dehydrogenase/reductase (SDR family) member 4	-2.000551	0.00554912
gene8228	GB48436	.	10.091832	0.00221603
gene8229	GB48344	G-protein coupled receptor moody	13.178946	1.4708E-06
gene8262	GB48331	GTP-binding protein RAD-like	-2.126168	0.00154127
gene8267	GB54260	probable 2-oxoglutarate dehydrogenase E1 component DHKTD1 homolog, mitochondrial	-2.083953	0.0014897
gene8305	.	uncharacterized LOC102655156	15.341224	2.3913E-05
gene8317	GB54226	unconventional myosin-IXb	2.685588	0.0002244
gene8325	GB54298	stromal cell-derived factor 2-like	-2.268797	1.2282E-06
gene8362	.	uncharacterized LOC102655726	-2.132831	0.0010987
gene8364	GB50876	uncharacterized LOC100578744	-2.286682	0.00078392
gene8377	GB50885	uncharacterized LOC409648	-3.087026	5.9114E-05
gene8384	.	uncharacterized LOC102654762	3.132497	0.00010526
gene8387	GB50891	solute carrier organic anion transporter family member 2A1	-2.730443	2.3677E-06
gene8406	GB51159	uncharacterized LOC413925	2.005570	0.00111021
gene8413	.	uncharacterized LOC107965032	2.603770	0.00069118
gene8414	GB51155	uncharacterized LOC726201	2.753282	0.00011649
gene8418	.	uncharacterized LOC102656499	5.855757	2.1636E-07
gene8484	.	zinc transporter 2-like	-2.213424	0.00073224
gene8489	GB50936	odorant binding protein 10	-2.098094	0.0001361
gene8506	GB50944	translocating chain-associated membrane protein 1-like 1	-2.419820	9.9623E-06
gene8513	GB50949	uncharacterized LOC408282	-2.005135	0.00144436
gene8524	GB51095	cryptochrome 2	-2.012087	0.00103934
gene8530	.	Z band alternatively spliced PDZ-motif protein 66	-2.069004	0.0008528
gene8580	.	uncharacterized LOC102654307	3.500907	1.6817E-05
gene8583	GB51063	uncharacterized LOC552276	2.034325	0.0005744

gene8630	GB51014	EF-hand domain-containing protein D2 homolog	9.274310	1.513E-06
gene8631	.	uncharacterized LOC107965113	2.099730	5.2077E-05
gene8642	GB48813	uncharacterized LOC408277	4.002738	0.03269999
gene8655	.	uncharacterized LOC102653912	2.189424	0.01413237
gene8682	.	uncharacterized LOC100578719	2.734002	5.9822E-06
gene8686	.	uncharacterized LOC107965036	-4.163160	2.0377E-05
gene8687	GB48784	mitochondrial cytochrome C	-2.915253	2.3651E-06
gene8730	.	abaecin	-2.227488	0.00094563
gene8763	.	uncharacterized LOC102654282	2.106016	0.00010217
gene8765	.	la-related protein 6	2.785790	0.01880276
gene8770	GB46652	peptidylprolyl isomerase B (cyclophilin B)	-2.237452	1.9066E-05
gene8778	GB46601	uncharacterized LOC724768	2.145454	0.00646763
gene8806	.	neuropeptide Y receptor-like	2.033534	0.00684134
gene8850	.	uncharacterized LOC107965170	2.089818	0.000394
gene8877	.	uncharacterized LOC102656589	2.082325	0.00044411
gene8878	.	uncharacterized LOC102656717	2.974179	4.3347E-05
gene8903	GB55205	major royal jelly protein 1	2.775429	7.8109E-06
gene8904	GB55206	major royal jelly protein 4	3.000317	0.00492088
gene8906	GB55207	major royal jelly protein 6	4.418685	5.555E-06
gene8907	GB55208	major royal jelly protein 5	2.103290	0.00012465
gene8921	GB55220	L-xylulose reductase	2.407545	0.00012398
gene8923	GB55164	lethal(2) giant larvae protein homolog 1	-2.447357	0.00019548
gene8924	.	uncharacterized LOC102655184	2.571526	0.01137217
gene8956	GB55144	dynein heavy chain 6, axonemal-like	2.656964	0.0001001
gene8963	.	uncharacterized LOC102654135	-4.345627	5.5769E-08
gene8991	.	uncharacterized LOC102653864	-2.360588	0.00148276
gene8994	GB55059	voltage-dependent T-type calcium channel subunit alpha-1G	-2.689348	0.0008041
gene9017	GB54056	serine hydroxymethyltransferase	2.081472	0.00073839
gene9069	GB53506	protein FAM49B	2.285158	0.00351608
gene9107	GB47278	sodium-independent sulfate anion transporter-like	3.103457	7.0569E-05
gene9108	GB47279	cytochrome P450 6k1	-3.657146	4.2821E-06
gene9116	.	uncharacterized LOC107965218	2.241863	0.00052815
gene9117	GB47285	uncharacterized LOC408348	6.332625	0.00012868
gene9118	.	uncharacterized LOC102656336	6.919692	5.1147E-05
gene9130	GB47301	UDP-glucuronosyltransferase 2C1-like	3.132103	0.00029741
gene9157	.	uncharacterized LOC102655369	4.201503	8.7873E-05

gene9263	GB45145	MAGUK p55 subfamily member 6	2.089043	0.00032887
gene9264	.	uncharacterized LOC102655046	4.925405	0.00438657
gene9265	.	uncharacterized LOC107965149	2.176239	0.00095735
gene9266	.	uncharacterized LOC107965150	2.240585	0.00080803
gene9278	GB45151	uncharacterized LOC408365	-3.321891	4.2984E-05
gene9298	GB45157	protein big brother	-3.615951	1.3018E-05
gene9335	GB44996	hexamerin 110	-2.591591	0.00026794
gene9340	.	uncharacterized LOC102656066	-3.128242	0.00018339
gene9374	GB44976	ataxin-2 homolog	2.570372	2.5077E-05
gene9425	.	apoptogenic protein 1, mitochondrial	-2.831451	0.00015076
gene9431	GB45211	troponin C type I	-2.289090	1.8936E-06
gene9434	.	myosuppressin	-3.382781	0.00301655
gene9470	.	uncharacterized LOC107965204	-4.400544	0.00010031
gene9471	.	uncharacterized LOC107965205	-2.509911	0.01843079
gene9472	.	uncharacterized LOC102653678	-3.189259	5.1203E-06
gene9488	.	uncharacterized LOC102654947	3.013025	0.01704573
gene9495	.	uncharacterized LOC102655371	2.656014	0.00013915
gene9590	GB44867	uncharacterized LOC100576985	5.363961	6.1013E-05
gene9616	GB43230	low-density lipoprotein receptor-related protein 2	-5.049410	0.00016769
gene9624	GB43225	putative inorganic phosphate cotransporter	2.071992	0.00280812
gene9625	GB43094	uncharacterized LOC726019	2.809513	0.00012237
gene9645	GB43214	dynein heavy chain 12, axonemal	2.192365	2.4551E-05
gene9649	GB43211	carbohydrate sulfotransferase 9-like	2.254586	0.00031913
gene9663	GB43205	uncharacterized LOC725391	-2.842104	2.2931E-05
gene9671	GB43201	allatostatin C	2.206377	0.0016996
gene9690	GB43130	single Ig IL-1-related receptor-like	2.084849	0.0031733
gene9692	GB43189	probable multidrug resistance-associated protein lethal(2)03659	-2.567258	2.9203E-05
gene9740	GB43168	inositol 1,4,5-triphosphate kinase	3.754242	5.8791E-07
gene9744	GB55730	CUGBP Elav-like family member 4	2.783952	5.961E-06
gene9761	GB40218	urea transporter 2-like	-11.067725	7.8114E-07
gene9763	GB40228	uncharacterized LOC100576488	-5.442279	0.00028703
gene9766	GB40231	collagen alpha chain CG42342-like	-2.295358	0.00014868
gene9791	GB40240	myosin regulatory light chain 2	-5.332578	0.00016902
gene9795	GB40198	odorant receptor 13a-like	-13.595296	1.146E-06
gene9796	GB40197	odorant receptor 13a-like	-14.484719	0.00020831

gene9813	GB51775	myosin heavy chain 95F	-2.541619	5.475E-06
gene9815	GB51782	carboxypeptidase Q-like	2.273434	0.00020207
gene9816	GB51783	carboxypeptidase Q-like	2.367622	0.00026103
gene9822	GB51787	myosin light chain alkali	-3.608456	8.3284E-05
gene9828	.	uncharacterized LOC107965329	-11.127903	0.0018841
gene9829	.	uncharacterized LOC102653740	-4.685359	0.00050804
gene9866	GB55063	dnaJ homolog subfamily C member 9-like	-2.446164	9.6677E-07
gene9887	GB53625	uncharacterized LOC411622	-18.563436	0.00012431
gene9928	GB40603	platelet binding protein GspB	-2.025832	7.7171E-05
gene9931	.	uncharacterized LOC107965382	-2.844218	0.00039407
gene9932	GB40606	uncharacterized LOC100577381	-4.112197	9.1503E-06
gene9935	.	proline-rich extensin-like protein EPR1	-3.175223	0.00014348
gene9936	GB40610	uncharacterized LOC100578276	-3.873352	8.96E-06
gene9937	GB40703	cadherin-23	-3.807018	1.4339E-05
gene9939	GB40614	vitamin K-dependent gamma-carboxylase	3.153446	1.5226E-07
gene9944	GB40619	troponin C type IIIa	-3.291405	1.1016E-06
gene9956	GB40697	apamin protein	-2.371599	0.0032866
gene9974	GB40635	venom acid phosphatase Acph-1-like	-4.557016	0.00023332
gene9986	.	uncharacterized LOC102655326	2.274719	3.2043E-06
gene9987	.	uncharacterized LOC107965308	2.336989	7.98E-05
gene10042	GB49306	ATP synthase, H+ transporting, mitochondrial F1 complex, gamma polypeptide 1	-2.248411	7.3114E-05
gene10051	.	uncharacterized LOC107965323	3.174751	1.9466E-05
gene10088	GB49328	uncharacterized LOC552110	-2.396586	3.9383E-05
gene10118	GB49347	prostaglandin reductase 1-like	2.662713	2.9548E-05
gene10149	GB52023	cytochrome P450 6AQ1	-2.091298	0.0001805
gene10154	GB52052	venom carboxylesterase-6	-3.060460	5.1475E-05
gene10160	GB52014	collagen alpha-1(I) chain-like	-2.457545	9.0758E-05
gene10214	GB52097	scavenger receptor class B member 1	-8.724390	0.00107003
gene10245	GB51967	ankyrin repeat domain-containing protein 6	-2.025168	0.00018457
gene10284	GB54767	zinc metalloproteinase nas-13	-2.403315	0.02005901
gene10336	.	uncharacterized LOC107965426	-2.944667	0.00072656
gene10340	GB48243	cyclin-dependent kinase 4	19.189850	2.3973E-06
gene10386	GB48216	troponin C type IIb	-6.408872	7.6123E-05
gene10391	GB50677	lipoma HMGIC fusion partner-like 2 protein	-2.753003	0.00043244
gene10439	GB47995	BMP and activin membrane-	-2.205850	0.0006159

		bound inhibitor homolog		
gene10446	GB47990	tropomyosin-1	-2.498255	0.00066391
gene10555	GB40168	6-phosphofructo-2-kinase/fructose-2,6-bisphosphatase	2.535182	0.000215
gene10576	GB53865	receptor-type guanylate cyclase gcy-4	3.506546	4.1378E-05
gene10616	GB49852	putative polypeptide N-acetylgalactosaminyltransferase 9	-2.638395	7.5651E-05
gene10647	.	uncharacterized LOC102655843	2.058902	0.0001504
gene10674	GB49890	cytochrome P450 6AS5	2.768433	5.9353E-06
gene10675	GB49885	probable cytochrome P450 6a17	2.364460	7.4183E-05
gene10758	GB48474	probable chitinase 3	-4.933703	6.452E-05
gene10805	GB42262	uncharacterized LOC100576738	-4.816805	6.2419E-06
gene10808	GB42277	ATP-dependent RNA helicase dbp2-like	-2.340875	0.00257508
gene10864	GB43575	trehalase-like	2.351334	2.1029E-06
gene10865	.	uncharacterized LOC102653891	-3.351023	1.1061E-05
gene10866	.	uncharacterized LOC100577863	-4.419062	0.00050688
gene10891	GB43560	insulin-like peptide 2	-2.306428	0.00158365
gene10945	GB43738	phenoloxidase subunit A3	2.021793	0.00024337
gene10946	GB43737	facilitated trehalose transporter Tret1-2 homolog	-2.736216	0.00361452
gene10963	.	cytochrome P450 9e2	-2.331485	0.00039113
gene10969	.	uncharacterized LOC107965484	2.035521	0.00018372
gene10985	GB54213	probable cation-transporting ATPase 13A3	-3.433994	0.00042042
gene11060	GB54172	sodium-independent sulfate anion transporter-like	-2.810920	7.4659E-05
gene11123	GB52756	apyrase	-2.228768	0.00023247
gene11130	GB52721	protein lozenge-like	3.841676	2.9965E-05
gene11132	GB52761	protein lozenge-like	2.168913	0.00010374
gene11138	GB52763	uncharacterized LOC100578776	3.037758	0.00156506
gene11139	.	uncharacterized LOC107965540	2.181985	6.2475E-05
gene11144	GB52767	chromatin assembly factor 1 subunit A-B	2.003848	0.00015386
gene11146	.	translation initiation factor IF-2-like	2.131686	0.00208274
gene11148	.	uncharacterized LOC725784	2.399502	7.1812E-06
gene11149	.	uncharacterized LOC100576925	4.105549	0.0002386
gene11169	GB52786	vesicle-associated membrane protein 2	-2.280052	0.00388572
gene11245	GB41651	glutamate dehydrogenase, mitochondrial	-2.364316	8.2437E-05
gene11287	GB41629	Eph receptor tyrosine kinase	-2.273353	0.00108173

gene11362	GB47075	uncharacterized LOC409185	-4.194239	3.029E-06
gene11366	.	ras-related and estrogen-regulated growth inhibitor-like protein	-2.033326	0.01322606
gene11375	GB41188	ABC transporter G family member 20-like	-3.058140	4.916E-05
gene11404	GB46217	cytoplasmic aconitate hydratase-like	2.215341	1.7428E-05
gene11426	GB46223	odorant binding protein 14	10.245410	0.00012565
gene11474	GB54417	farnesol dehydrogenase-like	-12.173741	4.7818E-06
gene11481	GB54664	multiple epidermal growth factor-like domains protein 10	3.107895	0.00016854
gene11503	GB54687	phosphate carrier protein, mitochondrial	-2.092876	0.00014476
gene11526	GB52910	octopamine receptor	-2.612552	0.00045109
gene11539	GB49476	lysyl oxidase homolog 4	-2.885570	1.8798E-05
gene11604	GB50550	uncharacterized LOC100576592	3.293930	3.9067E-05
gene11631	GB50573	uncharacterized LOC726094	2.848353	0.000263
gene11663	.	uncharacterized LOC102654477	2.896772	0.00026317
gene11685	GB50609	heat shock protein Hsp70Ab-like	2.695532	0.00038865
gene11686	.	.	2.452478	9.0976E-05
gene11691	GB50505	angiotensin-converting enzyme	2.910050	4.268E-05
gene11702	GB47932	headcase protein	2.234550	3.5186E-05
gene11705	GB47931	facilitated trehalose transporter Tret1-like	5.754616	0.00044589
gene11719	.	chymotrypsin inhibitor	3.643412	0.00251121
gene11720	.	cysteine-rich venom protein 1	3.755802	0.00137805
gene11725	GB50116	chymotrypsin inhibitor-like	-2.797833	0.01247092
gene11732	GB50123	myophilin	-3.410728	0.00035653
gene11757	GB50136	BCL2/adenovirus E1B 19 kDa protein-interacting protein 3	2.195257	9.0469E-05
gene11767	.	arrestin homolog	3.466319	7.0935E-05
gene11795	.	uncharacterized LOC102653894	2.015203	6.2322E-06
gene11826	GB50064	complexin	-2.077092	0.01606743
gene11836	GB50060	sulfotransferase family cytosolic 1B member 1-like	-2.864448	0.00016969
gene11851	GB50180	uncharacterized LOC552836	2.082354	0.00074246
gene11859	GB50184	uncharacterized LOC410600	2.018267	0.00125777
gene11863	GB50189	alpha-sarcoglycan	2.061741	0.00026522
gene11927	GB50012	uncharacterized LOC726323	4.957633	2.2382E-05
gene11937	GB50226	transferrin 1	2.512968	0.00010888
gene12016	.	uncharacterized LOC726965	2.160510	0.00308429
gene12019	.	uncharacterized LOC410581	-2.133795	4.0414E-05
gene12070	GB49937	PTS-dependent dihydroxyacetone kinase, dihydroxyacetone-binding	-2.017421	5.7042E-05

		subunit DhaK-like		
gene12093	.	uncharacterized LOC102655415	2.910090	2.9301E-05
gene12111	GB54396	elongation of very long chain fatty acids protein AAEL008004-like	-8.320193	1.582E-05
gene12124	GB54395	uncharacterized LOC413385	2.026865	0.00101534
gene12186	GB54350	spermatogenesis-associated protein 6	-2.147675	0.00025275
gene12192	GB54363	ATPase family AAA domain-containing protein 3	2.292249	0.0032788
gene12196	GB54356	growth factor receptor-bound protein 14-like	2.741723	0.00048745
gene12199	GB54361	metabotropic glutamate receptor	-5.000921	0.01487358
gene12245	.	uncharacterized LOC107965723	2.115259	0.00026205
gene12254	.	uncharacterized LOC102656308	2.106824	0.00062983
gene12260	.	uncharacterized LOC100577695	2.671390	4.369E-05
gene12264	.	uncharacterized LOC102656603	2.130556	7.6084E-05
gene12267	GB51283	retinal dehydrogenase 1	2.850396	0.00077957
gene12275	.	uncharacterized LOC102653684	3.304417	4.6625E-05
gene12276	GB51335	dihydrolipoyl dehydrogenase, mitochondrial	-2.047804	0.00023173
gene12305	.	uncharacterized LOC107965691	2.044803	0.01882709
gene12309	GB51356	cytochrome P450 4G11	2.501500	3.6074E-05
gene12312	GB51261	TWiK family of potassium channels protein 7-like	-2.054059	6.0463E-06
gene12398	.	insulin-like growth factor-binding protein complex acid labile subunit	-2.506791	0.00102021
gene12412	.	uncharacterized LOC100577317	-2.206467	0.00272629
gene12445	GB45609	flavin-containing monooxygenase FMO GS-OX-like 4	-2.059361	0.00170954
gene12453	GB45614	chymotrypsin inhibitor	-3.522875	0.00074934
gene12455	GB45951	yellow-f	-4.495286	4.1516E-06
gene12459	GB45955	beta-galactosidase-like	-2.038168	9.6239E-06
gene12472	GB45943	collagen alpha-5(IV) chain	-2.352315	4.3931E-05
gene12474	GB45968	collagen alpha-1(IV) chain	-3.112472	3.6354E-05
gene12481	GB45973	aromatic-L-amino-acid decarboxylase	3.792680	0.00011254
gene12534	.	.	-3.167605	9.864E-05
gene12536	GB48205	uncharacterized LOC727444	-4.427176	2.884E-05
gene12580	GB47902	endocuticle structural glycoprotein SgAbd-1	-3.558980	3.6504E-05
gene12585	GB47906	endocuticle structural glycoprotein ABD-4	-11.980619	0.00061604
gene12591	.	uncharacterized LOC100577556	-3.752655	2.7991E-05
gene12653	GB45746	probable cytochrome P450 6a13	-2.227748	3.6135E-05

gene12679	GB40877	translocon-associated protein subunit delta	-2.909555	3.3739E-05
gene12729	GB48510	serine protease 34	-2.880064	7.6711E-05
gene12735	.	uncharacterized LOC107965774	-2.265675	0.01006336
gene12764	GB54299	limbic system-associated membrane protein	-2.096400	0.01728401
gene12792	GB46973	uncharacterized LOC412222	-2.106465	8.4803E-06
gene12810	.	uncharacterized LOC102656256	-2.454098	0.00563809
gene12823	.	microRNA 3759	-5.089569	0.0014839
gene12835	GB46813	unconventional myosin-Ie-like	3.240991	9.1726E-05
gene12849	GB52658	general transcription factor 3C polypeptide 5	-2.468238	0.00042859
gene12850	GB52656	uncharacterized LOC552154	-4.295310	8.0112E-06
gene12858	GB17746	putative lipoyltransferase 2, mitochondrial	-2.769362	0.02326787
gene12897	GB55965	dehydrogenase/reductase SDR family member 4-like	-2.442749	2.6365E-05
gene12968	GB53270	UPF0428 protein CXorf56 homolog	-2.032052	0.00027673
gene12979	GB53286	uncharacterized LOC409402	2.531915	6.4137E-05
gene12984	GB55986	pancreatic triacylglycerol lipase	-3.833260	6.969E-06
gene13008	GB45919	organic cation transporter protein	-3.391306	0.00080441
gene13013	GB41015	glycine-rich cuticle protein	-4.309566	0.00108337
gene13078	GB50626	phospholipase D3-like	-3.343609	5.516E-05
gene13079	GB50627	putative fatty acyl-CoA reductase	3.037537	8.315E-07
gene13084	GB50622	voltage-gated potassium channel subunit beta-2	2.054123	0.00176702
gene13115	.	uncharacterized LOC102656271	-2.343414	0.01449895
gene13116	GB43353	uncharacterized LOC727578	2.720083	1.1229E-06
gene13139	GB49929	laminin subunit alpha	-2.070491	9.7391E-06
gene13141	GB49930	peptide transporter family 1	-5.172679	2.5107E-05
gene13150	.	uncharacterized LOC102654702	-2.383304	7.1234E-06
gene13188	.	selenium-binding protein 1-like	-2.198227	0.00181018
gene13194	.	uncharacterized LOC102656577	-2.058512	0.00617917
gene13230	GB42515	FAD-dependent oxidoreductase domain-containing protein 1	-2.090099	0.00102293
gene13245	GB50871	serine/threonine-protein kinase SIK2	-5.999554	7.7734E-07
gene13297	GB48193	acyl-CoA Delta(11) desaturase	-2.589854	0.0052068
gene13300	.	uncharacterized LOC100578075	2.496864	3.8798E-06
gene13304	GB42346	uncharacterized LOC100578206	-2.121159	0.010659
gene13320	GB52278	filamin-A-like	3.760396	0.00011548
gene13331	GB47132	voltage-gated potassium channel subunit beta-2-like	2.200931	1.9914E-05
gene13352	.	uncharacterized LOC102656820	3.164486	0.00999336

gene13358	.	uncharacterized LOC725449	12.447528	0.00015513
gene13369	GB45748	probable cytochrome P450 6a17	2.467323	0.00347688
gene13429	GB41110	uncharacterized LOC727546	-12.898643	6.8718E-05
gene13459	.	putative SLC9B1-like protein SLC9B1P1	-2.802851	0.00292934
gene13471	GB43056	catalase-like	2.411614	0.00013599
gene13473	GB43670	endocuticle structural glycoprotein SgAbd-8	-2.558103	0.00594383
gene13594	GB49819	branched-chain-amino-acid aminotransferase, cytosolic-like	-3.751388	2.7882E-05
gene13610	GB41007	dynein heavy chain 6, axonemal-like	2.452601	0.00182355
gene13630	.	3-hydroxy-3-methylglutaryl-coenzyme A reductase-like	-2.926283	6.9013E-05
gene13653	GB49662	uncharacterized LOC100578648	4.059510	0.00016984
gene13661	.	uncharacterized LOC102654849	-3.202050	0.00067189
gene13694	.	uncharacterized LOC102656237	-2.000002	0.00152702
gene13700	GB43350	catalase-like	2.528500	0.00018919
gene13755	.	uncharacterized LOC102655276	-2.233201	0.00342864
gene13763	GB47165	carboxypeptidase Q-like	2.195575	0.00101191
gene13803	GB49509	chymotrypsin inhibitor-like	-6.471307	0.0009525
gene13811	.	uncharacterized LOC102653942	-2.603805	0.00213423
gene13813	GB47520	uncharacterized LOC725305	-2.141844	0.00566504
gene13861	.	uncharacterized LOC102656002	-2.689892	0.00062286
gene13899	GB47536	sarcalumenin-like	-3.344862	0.00120188
gene13910	.	probable 2-oxoglutarate dehydrogenase E1 component DHKTD1 homolog, mitochondrial	-2.251044	0.00057705
gene13919	GB54490	.	-2.438109	9.6833E-05
gene13932	GB48492	take-out-like carrier protein	-3.628997	1.0388E-05
gene13935	.	uncharacterized LOC102655346	-2.083626	0.00083659
gene13939	GB40897	uncharacterized LOC100578351	3.272960	6.4452E-05
gene13963	GB53986	farnesol dehydrogenase-like	-14.170061	2.2214E-05
gene14038	GB43029	actin, indirect flight muscle-like	-2.811388	0.0035955
gene14052	GB42343	alanine--glyoxylate aminotransferase 2-like	4.301236	7.7942E-05
gene14065	.	ATP synthase F0 subunit 6	-2.568427	0.01367801
gene14066	.	cytochrome c oxidase subunit III	-2.056355	0.01679774
rna29219	.	.	-2.261926	0.02833222
rna29222	.	.	3.856199	0.00124267
rna29223	.	.	2.126902	1.3829E-05
rna29224	.	.	2.494478	0.00668994
rna29234	.	.	4.992673	0.00013884

rna29236	.	.	3.020381	0.00236858
