# Supplementary material for: Upregulation of Transferrin and Major Royal Jelly Proteins in the Spermathecal Fluid of Mated Honeybee (Apis mellifera) Queens
Source: Insects. 2021 Jul 31;12(8):690. doi: 10.3390/insects12080690 (PMC8396679; doi:10.3390/insects12080690)
Supplement: Supplementary file 1 [file insects-12-00690-s001.zip › Table S2.pdf]

Table S2. The lists of genes related to ontology and pathway in the spermathecae using the online tool DAVID.

Annotation	FDR	Gene list
Gene Ontology (Top 10 terms of DAVID functional annotation)		
Heme binding	0.000759418	GB46062, GB44808, GB45746, GB44513, GB45748, GB43056, GB49890, GB41427, GB55638, GB41406, GB52023, GB51356, GB48784, GB49885, GB46369
Structural constituent of cuticle	0.001871751	GB42581, GB43670, GB47906, GB47902, GB52161, GB52824, GB43353, GB46312, GB42612, GB46297, GB46311, GB50438
Monooxygenase activity	0.18318526	GB46062, GB45746, GB52023, GB51356, GB44513, GB49885, GB45748, GB49890
Iron ion binding	0.557327078	GB46062, GB45746, GB52023, GB51356, GB44513, GB49885, GB40967, GB55515, GB45748, GB49890
Oxidoreductase activity, acting on paired donors with incorporation or reduction of molecular oxygen	0.660356133	GB46062, GB45746, GB52023, GB51356, GB44513, GB49885, GB45748, GB49890
Calcium ion binding	2.001108598	GB55889, GB41301, GB49809, GB41806, GB48216, GB50700, GB51014, GB40240, GB52279, GB48552, GB51732, GB48576, GB53139, GB45211, GB40703, GB51787, GB40619
Pyridoxal phosphate binding	2.204567637	GB51583, GB47379, GB40442, GB42550, GB40451, GB49543, GB42343, GB45973, GB54056
Extracellular matrix structural constituent	4.781500738	GB52014, GB45943, GB45968, GB50700
Hydrolase activity, acting on ester bonds	23.44193618	GB41760, GB52756, GB42469, GB47668, GB42467

Transmembrane transporter activity	36.58030889	GB41034, GB45919, GB41126, GB48978, GB48382
Pathways (Top 10 terms of DAVID functional annotation)		
Tryptophan metabolism	0.042366006	GB51583, GB55638, GB41427, GB43350, GB49240, GB45973, GB43056, GB51283
Metabolic pathways	0.113795868	GB55499, GB41979, GB49937, GB48134, GB47379, GB49543, GB49240, GB51335, GB53579, GB45973, GB54056, GB55986, GB44430, GB48784, GB48078, GB17746, GB40451, GB46369, GB50626, GB49306, GB45834, GB49852, GB46217, GB45654, GB45538, GB53567, GB51583, GB45955, GB43168, GB48871, GB47503, GB40967, GB41651, GB55220, GB44491, GB51188, GB40336, GB55638, GB40734, GB47849, GB43247, GB49819, GB51591, GB43575, GB47301, GB51841, GB44367, GB48350, GB51283
Biosynthesis of antibiotics	0.221040796	GB53567, GB41979, GB51583, GB48134, GB43350, GB51335, GB49240, GB43056, GB54056, GB41427, GB44430, GB47849, GB40734, GB49819, GB51591, GB45834, GB46217, GB45538, GB51283
Carbon metabolism	0.577498775	GB49937, GB41427, GB43350, GB44430, GB40734, GB41651, GB40451, GB45834, GB51335, GB46217, GB45538, GB43056, GB54056
Gluconeogenesis	1.98469264	GB48134, GB40734, GB49240, GB45834, GB51841, GB51335,

		GB45538, GB51283
Biosynthesis of amino acids	2.420712369	GB53567, GB51583, GB40734, GB47849, GB49819, GB47503, GB40451, GB46217, GB54056
Glyoxylate and dicarboxylate metabolism	4.45311345	GB41427, GB43350, GB51335, GB46217, GB43056, GB54056
Oxocarboxylic acid metabolism	4.785491801	GB53567, GB51583, GB49819, GB40451, GB46217
ECM-receptor interaction	4.785491801	GB52014, GB45943, GB45968, GB49929, GB41126
Pentose and glucuronate interconversions	4.785491801	GB41979, GB47301, GB55220, GB49240, GB51283
