# Supplementary figures and images for: Upregulation of Transferrin and Major Royal Jelly Proteins in the Spermathecal Fluid of Mated Honeybee (Apis mellifera) Queens
Source: Insects. 2021 Jul 31;12(8):690. doi: 10.3390/insects12080690 (PMC8396679; doi:10.3390/insects12080690)

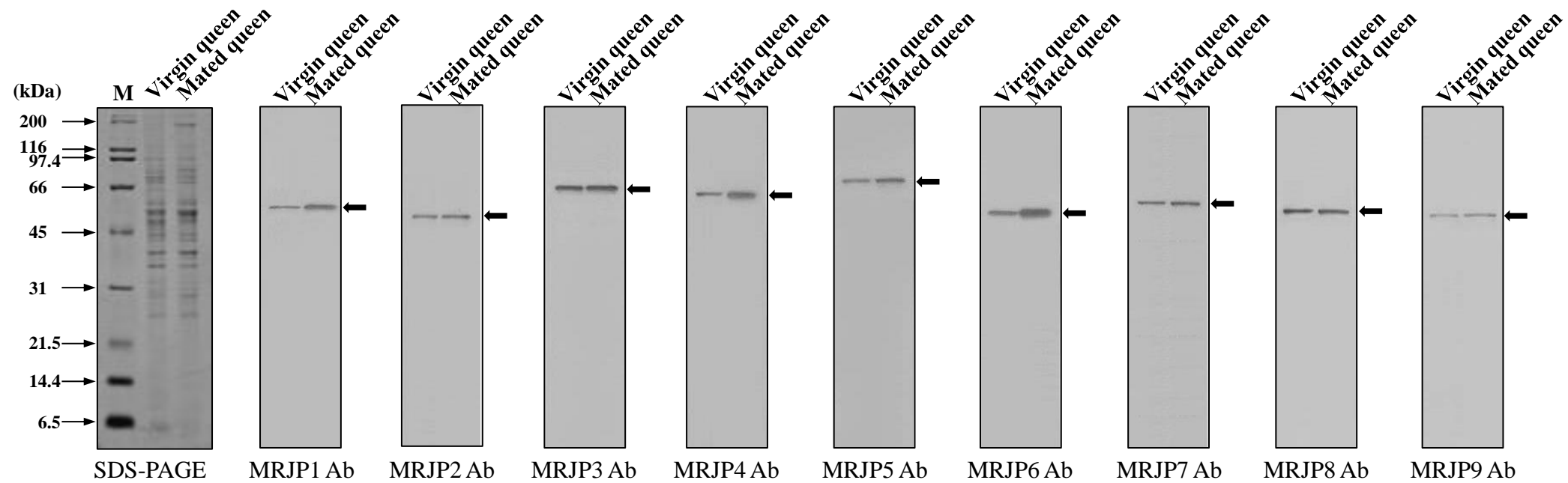

Supplement: Supplementary file 1 [file insects-12-00690-s001.zip › Figure S1.pdf]
